# Cancer patients’ intentions towards receiving unsolicited genetic information obtained using next-generation sequencing

**DOI:** 10.1007/s10689-017-0033-7

**Published:** 2017-08-29

**Authors:** Rhodé M. Bijlsma, Hester Wessels, Roel H. P. Wouters, Anne M. May, Margreet G. E. M. Ausems, Emile E. Voest, Annelien L. Bredenoord

**Affiliations:** 1 Department of Medical Oncology, Cancer Center, University Medical Center Utrecht, Q05.4.300, PO Box 85500, 3508 GA Utrecht, The Netherlands; 2 Department of Corporate Communications, University Medical Center Utrecht, Utrecht, The Netherlands; 3 Department of Medical Humanities, Julius Center, University Medical Center Utrecht, Utrecht, The Netherlands; 4 Julius Center for Health Sciences and Primary Care, University Medical Center Utrecht, Utrecht, The Netherlands; 5 Department of Genetics, University Medical Center Utrecht, Utrecht, The Netherlands; 6 Department of Medical Oncology, Netherlands Cancer Institute Antoni van Leeuwenhoek, Amsterdam, The Netherlands; 7 Department of Medical Humanities, Julius Center, University Medical Center Utrecht, Utrecht, The Netherlands

**Keywords:** Ethics, Genetics, Incidental finding, Unsolicited finding, Next-generation DNA sequencing, Cancer patients’ intentions, Needs and preferences, Theory of planned behaviour

## Abstract

Next-generation sequencing (NGS) can be used to generate information about a patient’s tumour and personal genome. This powerful diagnostic tool provides solicited and unsolicited hereditary genetic (risk) information that could have consequences for cancer patients and their quality of life. A well-defined approach for returning appropriate genetic risk information is needed in personalized cancer care. A qualitative design with semi-structured interviews was used. We conducted interviews with 24 Dutch patients with different types of cancer, both NGS-experienced and NGS-inexperienced, to learn their intentions, needs and preferences towards receiving unsolicited genetic information obtained using NGS. Almost all participants had a positive attitude towards receiving unsolicited findings. After receiving comprehensive background information on NGS, including a binning model of four categories of unsolicited findings, most participants preferred to receive only subsets of genetic information. Their main concern was their own and others’ (including family members) ability to cope with (the increased risk of having) a genetic disorder. Providing background information gave cancer patients the opportunity to select subsets of findings and increased their ability to make an informed choice. Special attention is needed for social and emotional factors to support the patients themselves and when communicating test results with their family members.

## Background

Today, systemic cancer treatment decisions are based not only on the tissue of origin, but also increasingly on genetic information. Mapping the genetic sequence of tumours in individual patients is expected to become a central feature in personalised cancer care.

Next-generation sequencing (NGS) technologies enable the affordable sequencing of whole genomes within a short timeframe. This powerful diagnostic tool can be used to generate solicited and unsolicited hereditary genetic (risk) information that could have medical, psychological, financial and social consequences for patients and a considerable impact on their quality of life [1, 2]. A well-defined approach for returning genetic risk information to cancer patients and their family members is therefore needed.

We [3–5] and others [6, 7] have developed disclosure policies for the feedback of genetic information in the context of large-scale genetic testing [8, 9]. Some of these policies consist of tiered consent models, where genetic results are offered in categories of genetic mutations, also known as binning models [1, 2]. Earlier empirical, often US-based, studies confirmed patients’ and research participants’ preferences to have results returned [3, 10–14]. A few studies have specifically focused on cancer patients’ preferences [15–18]. One of these tested a binning model, presenting six different types of individual genome sequencing results to a selected group of young breast cancer patients, who were found to be primarily interested in receiving information about actionable mutations [16].

We earlier described the occurrence of unsolicited findings in a Dutch research setting [19]. Further research is needed to examine the preferences of cancer populations; therefore, we conducted interviews with 24 Dutch patients with different types of cancer, who included patients with or without previous NGS experience (NGS-experienced and NGS-inexperienced, respectively), to learn their intentions, needs and preferences towards receiving unsolicited genetic information obtained using NGS.

## Methods

### Design

A qualitative design using semi-structured interviews was used.

### Participants

A total of 24 Dutch patients with different types of cancer, both NGS-experienced and NGS-inexperienced, were recruited by their oncologists. The main inclusion criteria were that they were 18 years old or older and had received a cancer diagnosis (any origin and any stage of disease). Patients unable to speak, read or write the Dutch language were excluded from the study.

Participants who had previously experienced a NGS procedure were informed by an investigator about the aims of that previous study, the related procedures, and also about the possibility of discovering unsolicited genetic findings [19]. After the patient had received a reasonable period to consider study participation, those willing to participate signed an informed consent form, which included a paragraph addressing the possibility of discovering unsolicited findings. To complete the informed consent form, patients had to explicitly answer questions about whether they wanted to receive unsolicited findings.

We appoint these patients ‘NGS-experienced’, meaning that these participants intended to be candidate for future anticancer treatment by participating in a trial that included NGS of both somatic and germline DNA. Hence, the NGS-experienced participants already underwent a tumour biopsy for sequencing reasons and were familiar with the possibility of revealing unsolicited findings during the sequencing and also with the possible need for a referral to a clinical geneticist.

### Semi-structured interviews

For the semi-structured interviews, an interview guide was developed and pre-tested. We adapted the surveys used in the ClinSeq study [11, 20] and added questions concerning, for instance, perceived behavioural control and questions about patient needs and preferences regarding education and counselling when learning the results of NGS.

Our interview guide was based on the health-related theory of planned behaviour (TPB), following the guidelines of Ajzen [21]. This theory integrates a person’s intentions to perform a specific behaviour, including their attitudes towards the behaviour, subjective norms, and perceived behavioural control. These intentions account for considerable variance in actual behaviour. The behaviour examined in this study was ‘making a decision on receiving information about unsolicited findings from NGS’.

The research protocol was approved by the Research Ethics Committee (IRB) of the University Medical Center (UMC) Utrecht (The Netherlands), and written informed consent was obtained from all participants.

### Interview procedure

**Fig. 1:**
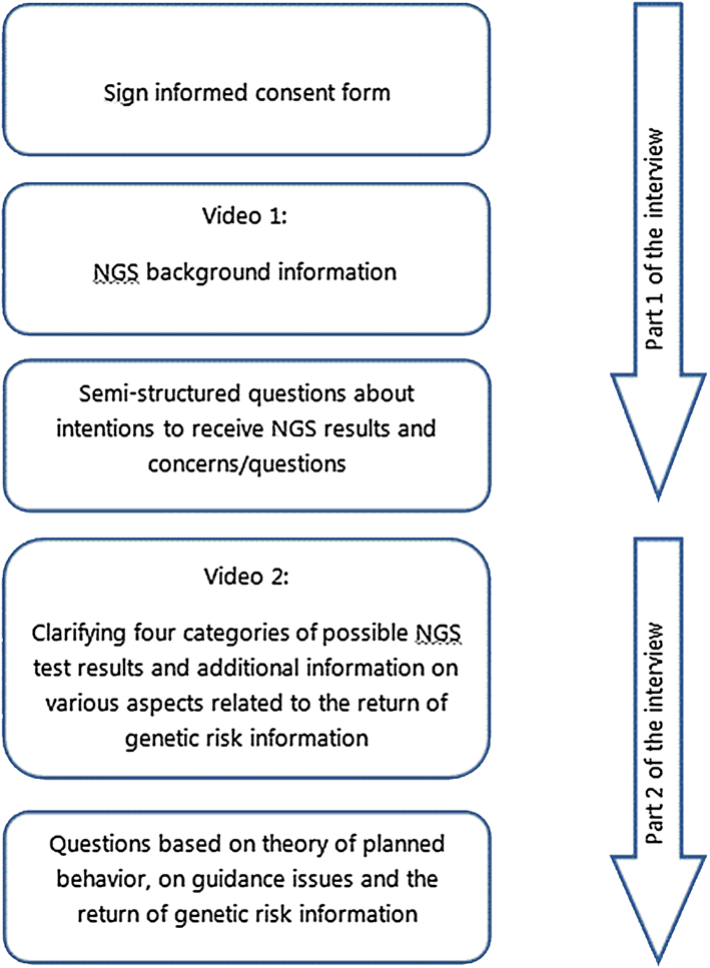
Interview strategy

Individual in-depth interviews took place at the UMC Utrecht Cancer Center. The interviews consisted of two parts and we showed patients two videos. The videos were used to ensure that all participants had received the same level of information on genome sequencing. Figure 1 shows our interview strategy.

The videos were in Dutch and were developed by the authors. The first video provided each participant with the same background information about NGS procedures, for example why NGS is medically useful and to explain the possibility of generating unsolicited genetic information. After this video, we asked the participants for an initial response, particularly for their preferences on whether they would like to be informed about unsolicited findings. We also determined whether they had any concerns or additional remarks on this topic. Table 1 shows our semi-structured interview guide for part 1 of the interview.

**Table 1:** Semi-structured interview guide of part 1 of the interview

Would you like to be informed about unsolicited findings? Yes/No/other
What is the reason that you would or would not want to be informed about unsolicited findings?
What are reasons for having the intention to receive or not to receive information about unsolicited findings?
What do you see as advantage or disadvantage of being informed about unsolicited findings?
Are there persons in your vicinity who would approve of your desire to be informed about the unsolicited findings?
Are there persons in your vicinity who would disapprove of your desire to be informed about the unsolicited findings?
Are there any factors or circumstances that make it difficult or impossible for you to be informed about unexpected results?
What questions come to your mind when you think about this topic?
Are there any concerns when you think about this topic?
What additional information do you need to make an informed decision about whether or not wanting to be informed about the unsolicited findings?

**Fig. 2:**
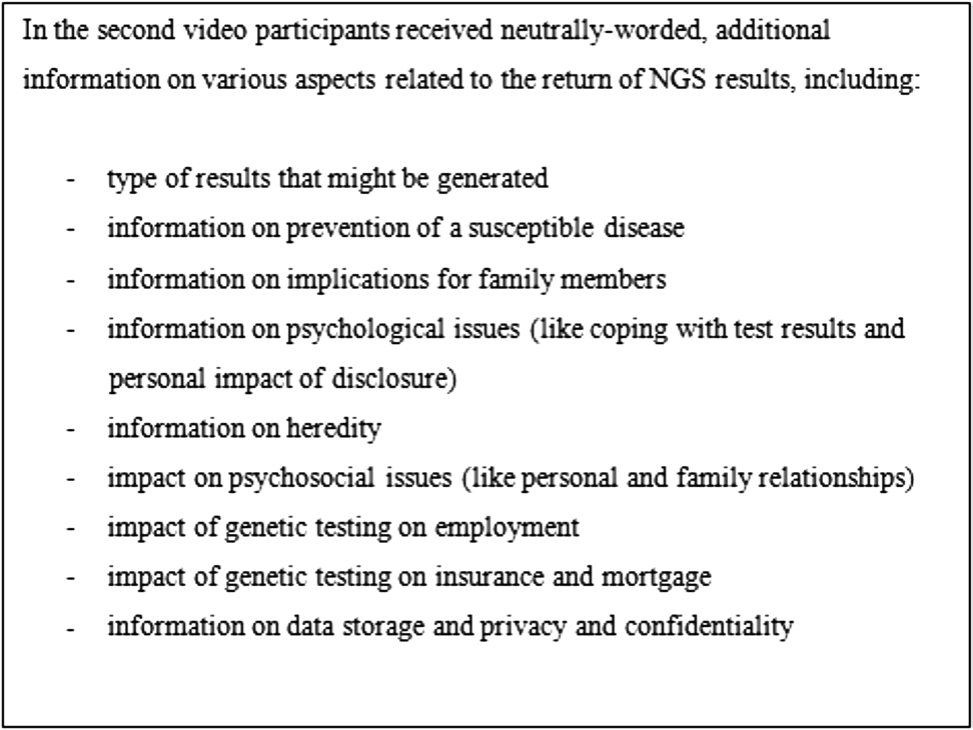
Topics discussed in video 2

In the second video, participants received information on various aspects related to the return of NGS results, as described in Fig. 2. The investigators offered participants time to consider the information they had learned from the second video and asked all participants whether they had any questions.

They also learned about four distinct categories (“*bins*”) of genetic information, shown in Table 2. These categories were based on our previously developed qualified disclosure policy [3, 4, 11].

**Table 2:** Four categories of genetic test results

Category 1	Category 2	Category 3	Category 4
A gene variant that predisposes you to a disease that can be prevented or treated	A gene variant that predisposes you to a disease that cannot be prevented or for which no current effective treatment has been established yet	A gene variant that does not affect your health, but that may be important to the health of your other relatives, such as your children or future offspring	Uncertain gene variants, meaning they may or may not be important to your health or the health of your relatives
Example: you have a gene variant which means you are much more likely to develop breast cancer. In this case, we may recommend that you more closely monitor your breasts or have prophylactic surgery	Example: you have a gene variant which implies that you are more likely to develop Alzheimer’s disease. Alzheimer’s disease cannot be treated or prevented	Example: you could learn that you have a variant in the gene that may cause Cystic Fibrosis (CF) in future offspring if the father would have this variant in his gene too	Example: you have a so-called unclassified variant, which implies you do have a variant for example for an increased risk of breast cancer, but the significance is unknown

In the second part of the interview, we used our binning model to present four distinct categories of genetic test results, asking questions based on TPB [21].

### Data analysis

The interviews were audiotaped and transcribed verbatim. Data analysis was undertaken using the constant comparative method, which involves going back and forth from the data to develop codes, concepts and themes [22, 23]. RB independently coded the full transcripts by labelling units of texts that referred to one or more topics relevant to the study’s aim. Coding was done with NVivo 10 software. HW and ALB read the full coded transcripts and checked the codes for consistency. The codes were adjusted by comparison across transcripts and following discussion with the other authors. The coding outline was modified and transcripts were re-analysed.

## Results

In total, 24 interviews were conducted between April 2014 and December 2014 by RB in the presence of HW, who made field notes during the interviews. Data saturation was reached after 21 interviews. In the last three interviews, it was confirmed that no new thematic content was found, and after interview 24 the recruitment was ended [24]. Each interview lasted approximately 1 h.

The interviews were conducted with seven participants with curable-stage disease and 17 with advanced-stage disease. From the advanced-stage cancer patient group, eight participants had previously had NGS performed on their tumours. All other patients were NGS inexperienced.

The majority of our participants were Caucasian. Participants were, on average, 60 years of age (29–79 years) and had a high level of education. Table 3 shows the patient characteristics.

**Table 3:** Patient characteristics

Patients n=24	Total
Age 29–79 years	
≤55 years old	10 (42%)
>55 years old	14 (58%)
Gender	
Male	13 (54%)
Female	11 (46%)
Stage	
Curative, NGS inexperienced	7 (29%)
Advanced stage, NGS inexperienced	9 (38%)
Advanced stage, NGS experienced	8 (33%)
Education level	
Low	3 (13%)
Medium	5 (21%)
High	16 (66%)
Diagnosis	
Brain tumor	2
Breast cancer	5
Cholangiocarcinoma	1
Colon Carcinoma	1
Epithelioid hemangio endothelioma	1
Larynx carcinoma	1
Melanoma	2
Ovarian cancer	2
Pancreatic cancer	1
Prostate cancer	3
Renal cell carcinoma	1
Testicular cancer	4

### Attitude and intention

In line with the TPB, we invited cancer patients to think about their intentions towards receiving unsolicited genetic information. For our participants this was a hypothetical situation, as no real-life data were available to return to them. Most participants had a positive attitude towards receiving NGS results. At the start of the interviews, almost all participants, both curable- and advanced-stage, wanted to receive all available genetic information.

The intention to receive unsolicited findings changed during the interview. Initially, most participants indicated that they preferred to be informed about all possible genetic findings arising from NGS; however, after the second video, which introduced the four categories of genetic test results, more than half of our participants favoured limiting feedback to one or more subsets of genetic variants.

### Motivations for receiving unsolicited genetic information

When asked about their motivations for receiving unsolicited genetic findings, some of the NGS-experienced patients stated that they had participated in a sequencing procedure to contribute to the advancement of medical science in cancer treatment. These participants probably meant that they accepted the outcome of NGS as a package deal consisting of cancer-related personal treatment possibilities and possible unsolicited findings. Interviewer: “Do you want to receive unsolicited findings?”. Respondent: “Of course. I think medical science could develop more targeted tools” (Male, 52, advanced stage, NGS experienced). Another participant answered this question with: “Yes, for medical science” (Male, 78, advanced stage, NGS experienced).

Others stressed the importance of contributing to future healthcare from an economic point of view, by saving medication for those patients that would profit from a NGS-discovered mutation that could be targeted by anti-cancer treatment: “The drug use and chemo treatments could perhaps then be even more specific” (Female, 67, advanced stage, NGS inexperienced) and “The right resources in the right place” (Male, 72, advanced stage, NGS experienced).

Most participants explicitly mentioned that they would like to receive unsolicited findings simply for their own interest: “I did not need any motivation, I just did it for myself” (Male, 72, advanced stage, NGS experienced).

Almost all participants, regardless of having curable- or advanced-staged cancer, indicated that they would be willing to adapt their lifestyle towards healthier behaviour or to undergo screening or (preventive) surgery to decrease their future disease risk. Our participants expressed their wish to be prepared for possible future diseases: “So I can make some adjustments to my lifestyle (…) to prevent or to reduce the chance” (Male, 37, curative stage, NGS inexperienced).

Some participants indicated that they would like to be informed about unsolicited findings to help their close family members, with some feeling responsibility towards their children: “I would like to know if there are consequences (…), for me personally, especially for my children, and also for first- and second-degree family members, anything that they need to know” (Female, 57, advanced stage, NGS inexperienced).

### Conditions for receiving unsolicited findings

Regularly, participants spontaneously added conditions to be met before they would willingly receive unsolicited findings; for example, the condition only to receive unsolicited information if the disease would manifest itself in the short term. Participants wanted to be informed about conditions to which they were susceptible, the probability of developing them, expressed in percentages, the consequences of the disease and the availability of preventive measures: “I would like to know more concretely which diseases we are talking about and what are their consequences (….),then I would like to know the likelihood of getting these diseases. I would then prefer to know (…) whether there is a possible form of prevention, or if something can be done with the knowledge, for example, making lifestyle adjustments” (Female, 57, advanced stage, NGS inexperienced).

Several participants reported that they only wanted to receive information if there was an opportunity to influence the course of a susceptible disease: “If you can do something to reduce the outcome of the disease, for yourself or the family” (Female, 67, advanced stage, NGS inexperienced). “If there is a high probability, for example 70%, that I could get Alzheimer’s disease, then for me the information is of limited use” (Male, 37, curative stage, NGS inexperienced).

### Reasons why participants do not want to receive unsolicited findings

Although the majority of participants said they wanted to receive unsolicited findings, there were also some who were reluctant to receive this genetic information. The main reasons given were concerns about their own or others’ ability to cope with a genetic disorder and the emotional burden they expect upon receiving this information. They also mentioned that they did not want to upset their family members: “The downside is (…) that it can make you very depressed (…). I find it quite challenging to upset my children with particular information when it comes to genetic disorders” (Female, 66, advanced stage, NGS inexperienced).

A few participants referred to insurance and privacy issues as reasons not to receive NGS results. Some participants stated they would like to remain ignorant of the possible return of their cancer in the future. “I think it is very hard to explain to people who have not had cancer. It seems like something you just live with in your mind, but you would have known this for all these years” (Female, 52, curative stage, NGS inexperienced).

### Subjective norm

We asked patients whether specific individuals or groups in their personal lives would encourage or discourage them to receive unsolicited genetic findings. Almost all participants indicated that their family members and relatives would encourage them to receive the information. “My wife, like my children, encouraged me” (Male, 78, advanced staged, NGS experienced). A few participants stated that they did not care what other people recommended, and a few participants knew a relative would be reluctant to see them receiving unsolicited findings.

### Perceived behavioural control

Cancer patients could be concerned about the barriers to making a decision to receive information about unsolicited NGS findings, particularly the anticipated emotional burden. These barriers involve the cancer patients themselves or their family members, particularly their children. In this context, they mentioned concerns about the heredity of their cancer: “I immediately think about my children. I hope that they do not have the same genetic abnormality*”* (Female, 69, advanced stage, NGS experienced).

Participants expressed concerns for the near future: “The art of not knowing is that you have to deal with it very consciously (…). What is the consequence of knowing the unsolicited results and what is the consequence of not knowing?” (Male, 52, advanced stage, NGS experienced). Although participants discussed the impact on their families, almost all stated that the ultimate decision to receive unsolicited findings in the future is entirely up to themselves.

One aspect facilitating the participants’ ability to make a choice about whether to receive unsolicited findings was the possibility for them to adapt their lifestyle when an increased susceptibility to a specific disease was identified. The cancer patients considered the options to undergo screening or (preventive) surgery to decrease their future disease risk, as described in ‘Motivations for receiving unsolicited genetic information’.

### Needs and preferences in education and counselling

To be able to make informed decisions, patients expressed several needs and preferences concerning education and counselling during the process of NGS.

Patients indicated their need to be supported when communicating the unsolicited genetic information to family members. “How can I communicate the information to the people it concerns?” (Male, 52, advanced stage, NGS experienced). Furthermore, patients expressed a need for written background information on the unsolicited finding: “(I need) accessible, written information” (Male, 74, advanced stage, NGS inexperienced), as well as the need of psychosocial assistance on demand: “I wish that psychosocial support was offered, and that this support was still available even years later” (Female, 57, advanced stage, NGS inexperienced).

Several participants asked for a period to decide whether they wanted to receive the unsolicited information: “I can imagine a kind of ‘waiting time’, for example two weeks, to consider whether I really want these results” (Female, 52, curative stage, NGS inexperienced). Another participant would like to involve his family doctor, by giving him a sealed envelope that at some point in time could be opened to share the unsolicited information (Male, 37, curative stage, NGS inexperienced).

## Discussion

The behaviour examined in this study using TPB [21] was ‘making a decision on receiving information about unsolicited findings from NGS’. Consistent with the literature [3, 10, 11, 13], the attitude of most of our participants, both curable- and advanced-stage, NGS-experienced and NGS-inexperienced cancer patients, was positive towards receiving (unsolicited) genetic information from NGS performed during cancer diagnosis. After more background information on the NGS procedure and the various aspects related to returning the results was provided, including the four categories of unsolicited findings, our participants became more conservative and seemed to be more aware of the possible consequences of receiving genetic risk information. They adjusted their answers to receive only subsets of information instead of all genetic variations. This is in line with findings of Bollinger et al. [12], who showed that patients change their preferences regarding the disclosure of unsolicited findings after discussing different types of results. Other quantitative studies in cancer patients [25] and healthy persons [26] have also confirmed that study participants provide more nuanced answers when given more background information. This underscores the importance of providing adequate information and counselling. Receiving (written) information was previously described as a tool to reduce patients’ anxiety [27], and as being reassuring [28]. Written information facilitates better understanding and decision making and can also help patients to communicate genetic information to their families [29]. Receiving unsolicited findings in person, from a medical professional or genetic counsellor or geneticist, could help to explain the results and their implications [15, 16].

Participants in our study expressed clear conditions for receiving unsolicited findings, such as information about the probability of developing a particular condition (expressed in percentages) and the availability of preventive measures for the diseases that could be revealed.

The patients showed interest in gaining knowledge about their health and body, as well as information on how to prevent future diseases. They suggested that receiving genetic insights would give them the opportunity to prepare their personal lives and, if necessary, make health-related lifestyle adjustments. Although they discussed their personal social environment in detail, patients declared that the final decision to receive unsolicited risk information was completely their own. This question was explicitly asked to every participant for all four categories of results (outlined in Table 1). For each category, almost every patient indicated that they wanted to make the ultimate decision on receiving information by themselves. This subjective norm result is notable and worthy of further investigation, given the inherent familial nature of genetic results.

In order to understand how the grouping of genetic information into multiple categories may support patients to sustain or improve their health, we will use the concept of health as introduced by Huber et al. in 2011 [30]: “Health as the ability to adapt and to self-manage, in the face of mental, social and physical challenges”. This concept describes health not as a stable endpoint, as in the traditional WHO definition, but highlights function, resilience and self-direction. This definition is useful in this context for several reasons. First, it focusses on the patient’s capability to cope with health conditions rather than on the actual impairments. In our study, participants changed their preferences after they encountered the possibility of receiving categories of genetic information; moreover, patients valued the opportunity to choose between packages. Applied to this definition, distinguishing between categories of diseases gives patients the opportunity to select a subset of findings, which might increase their ability to deal with unsolicited genetic information. Clinically actionable findings might enable these patients to lead a healthier life and consider themselves as healthy, while other findings (e.g. incurable diseases) emphasise their inabilities and therefore make them feel ill. Second, Huber and colleagues acknowledge the importance of social factors as constitutive features of health. We have shown, using the TPB, that the attitudes of family members, doctors and other important persons influence the patients’ intentions towards receiving genetic findings. Third, the finding that perceived behavioural control influences the way patients perceive genetic information also fits within Huber’s definition. Anticipated psychological stress, either their own or in relatives, changes patient perspective on the feedback of unsolicited findings.

### Study limitations

Our study has some limitations. Most participants were Caucasian, highly educated, and all were recruited from a single (though large) Dutch academic hospital; therefore, the results may not be generalised to other patient populations. Further, our participants might have been particularly interested in DNA sequencing or might be familiar with NGS procedures, for example due to previous procedures. In addition, the feedback of unsolicited findings was presented as a hypothetical situation, as none of the participants with NGS experience had received an unsolicited finding.

Despite offering information as comprehensibly as possible, it became clear during the interviews and the subsequent analysis that this is a complex topic, and that some participants had difficulties differentiating between the aims of a NGS procedure and the research question concerning the return of unsolicited findings.

Comparing participants that had actually undergone NGS with those who were NGS inexperienced could provide interesting insights, as could the comparison between curative- and advanced-stage cancer patients, or determining the differences between younger patients of childbearing age versus older patients. However, a qualitative study typically has a relatively small sample size which means that we were not able to generalise the results of our semi-structured interviews into different subgroups of participants. More quantitative research is needed to examine the feedback of unsolicited findings in larger groups to better explore differences between these patients. Based on our qualitative study, we are now setting up quantitative research that will focus on a larger group of cancer patients.

### Clinical implications for daily practice

During the use of NGS in clinical practice, education and counselling is vital to enable patients to make an informed choice. Presenting categories of genetic test results was found to be a useful tool in enabling cancer patients to make a well-informed decision about receiving unsolicited findings from NGS. Like other patient groups, our cancer patients adjusted their answers after receiving more background information. They seemed to be more aware of the possible consequences and choose to receive only subsets of information instead of all genetic variations linked to disease.

Special attention must be given to the social and emotional factors needed to support the patients themselves as well as their communication of the test results with their family members. Also, an important point for healthcare professionals to acknowledge is the fact that this topic is rather difficult to understand, even for highly educated patients.

The results of our study emphasise the importance of providing tailored information related to the return of NGS information. We highlight the importance of supporting healthcare professionals in the education and counselling of patients when communicating unsolicited results in the context of personalised cancer care and NGS. A decision aid should be developed to optimally support cancer patients.
